# Contacts and behaviours of university students during the COVID-19 pandemic at the start of the 2020/2021 academic year

**DOI:** 10.1038/s41598-021-91156-9

**Published:** 2021-06-03

**Authors:** Emily Nixon, Adam Trickey, Hannah Christensen, Adam Finn, Amy Thomas, Caroline Relton, Clara Montgomery, Gibran Hemani, Jane Metz, Josephine G. Walker, Katy Turner, Rachel Kwiatkowska, Sarah Sauchelli, Leon Danon, Ellen Brooks-Pollock

**Affiliations:** 1 School of Biological Sciences, University of Bristol, Bristol Life Sciences Building, 24 Tyndall Avenue, Bristol, BS8 1TQ UK; 2 Population Health Sciences, University of Bristol, Bristol, UK; 3 NIHR Health Protection Research Unit in Behavioural Science and Evaluation, University of Bristol, Bristol, UK; 4 School of Cellular and Molecular Medicine, University of Bristol, Bristol, UK; 5 Bristol Veterinary School, University of Bristol, Bristol, UK; 6 Bristol Children’s Vaccine Centre, University of Bristol, Bristol, UK; 7 National Institute for Health Research Bristol Biomedical Research Centre, University Hospitals of Bristol, Weston NHS Foundation Trust, University of Bristol, Bristol, UK; 8 Department of Engineering Mathematics, University of Bristol, Bristol, UK BS8 1TW; 9 Alan Turing Institute, British Library, London, UK

**Keywords:** Infectious diseases, Viral infection, Human behaviour

## Abstract

University students have unique living, learning and social arrangements which may have implications for infectious disease transmission. To address this data gap, we created CONQUEST (COroNavirus QUESTionnaire), a longitudinal online survey of contacts, behaviour, and COVID-19 symptoms for University of Bristol (UoB) staff/students. Here, we analyse results from 740 students providing 1261 unique records from the start of the 2020/2021 academic year (14/09/2020–01/11/2020), where COVID-19 outbreaks led to the self-isolation of all students in some halls of residences. Although most students reported lower daily contacts than in pre-COVID-19 studies, there was heterogeneity, with some reporting many (median = 2, mean = 6.1, standard deviation = 15.0; 8% had ≥ 20 contacts). Around 40% of students’ contacts were with individuals external to the university, indicating potential for transmission to non-students/staff. Only 61% of those reporting cardinal symptoms in the past week self-isolated, although 99% with a positive COVID-19 test during the 2 weeks before survey completion had self-isolated within the last week. Some students who self-isolated had many contacts (mean = 4.3, standard deviation = 10.6). Our results provide context to the COVID-19 outbreaks seen in universities and are available for modelling future outbreaks and informing policy.

## Introduction

By November 2020, the COVID-19 pandemic had caused 1.2 million deaths globally^1^ and in many countries had forced the temporary closure of educational institutions, including universities^2^. In the Autumn of 2020, with reported daily COVID-19 cases rising nationally^3^, students at UK universities began to return for the start of the 2020/2021 term. Whilst university students, due to their age, are less affected by COVID-19 morbidity and mortality than other groups^4^, up to one third still may be medically vulnerable to severe COVID-19^5^ and all infected students still have the potential to transmit the virus to others. University students often travel from across the country and the globe to their place of education and have the potential to facilitate onward transmission of infection carried from their home locations. In addition to the national COVID-19 restrictions in place during Autumn 2020, UK universities implemented a range of measures to reduce transmission such as reducing the amount of in-person teaching through delivery of lectures online and restricting student living circles^6^. However, despite these measures, large outbreaks of COVID-19 occurred across many UK universities^6,7^.

At the University of Bristol (UoB), there was an online induction week from 28th September to the 2nd October and the first teaching block started on the 30th September. The UoB adopted a “blended” teaching approach based upon a mixture of in-person and online teaching. In university-owned halls of residence, students were divided into households (“living circles”) ranging from 1 to 44 individuals per household (median = 5, interquartile range [IQR]: 1–7)^8^. Students were instructed not to host any non-residents in their household but could meet others outside of their household provided they conformed to the government social distancing guidelines and other relevant infection control measures such as use of face coverings where appropriate to do so^9^.

The UoB reported positive test results daily since the 14th October 2020^10^, with 1722 positive tests among UoB students being reported up until the 1st November, roughly 7% of students, compared with 48 positive tests among staff (< 1%) over the same period. On the 9th October, 300 students in one University-owned hall of residence were requested to begin a 14-day period of mass self-isolation^11^ and then on the 13th October an additional 40 students in a block of four flats in a separate location were also asked to start a 14-day period of self-isolation^12^. The vast majority of students living in these large halls of residence are first year undergraduates^13^. Students that tested positive in other accommodation types were required to isolate along with their household, in line with national guidelines^10^.

Although there have been previous studies prior to the COVID-19 pandemic that have collected data on contact patterns^14–17^, only a small sample of these have been relevant to students^14,15^ or participants could not be identified as students^16,17^. Furthermore, the behaviour of students may have changed in view of the pandemic and in response to government regulations. During the pandemic, the CoMix social contacts survey has been collecting data on contact patterns in the general UK population^18^, however, there have been no specific reports on students. Understanding contact patterns, COVID-19 related symptoms and behaviour of students is important to inform public health action and mathematical models. Here, we aim to fill this knowledge gap and quantify the behaviours and contact patterns among students of the UoB during the start of the 2020/2021 academic term by carrying out an online survey.

## Methods

CONQUEST (COroNavirus QUESTionnaire) is an ongoing online survey on contacts, behaviour, and potential SARS-Cov-2 symptoms for staff and students at UoB. This survey has been live since the 23rd June 2020. Participants complete an initial questionnaire which include questions on background demographics and then are given the option to fill out a shorter version of the questionnaire on contacts, symptoms, and whether they have had COVID-19; repeating this every 8 days. Initially there was high participation from staff members, but very low participation from students, principally because the survey was launched near the end of the 2019/2020 academic year when most students had returned home. From the start of the 2020/2021 academic year, there were several initiatives to recruit more students to complete the survey (see Supplementary materials). Here, we present a subset of the survey data from the 14th September 2020 to the 1st November 2020, mainly focusing on the student data.

### Survey

The survey data were collected and managed using REDCap Electronic Data Capture tools hosted at the UoB^19,20^. The full questionnaire has been provided in the Supplementary materials. The survey captured demographic information, information about participants’ contacts on the previous day, information about symptoms during the previous week, whether participants had been self-isolating during the previous week, and COVID-19 status if known.

Demographic information on participants was captured when they completed the initial survey. This included data on age, gender, ethnicity, whether they were part of a high-risk group, whether they were a student, a member of staff, or both, whether they were an undergraduate or postgraduate, their study year, their UoB department, their residence, and the age of their household members.

Participants were asked about three types of contacts they had had on the previous day:

1. Individual contacts—those who they spoke to in person one-on-one, including those in their household and support bubble.
2. Other contacts—if they spoke in person to many people one-on-one in the same setting (but they did not have the opportunity to speak to each other), for example, as part of working in a customer service role in a shop.
3. Group contacts—large groups of individuals in the same setting (for example, sports teams, tutorials, lectures, religious services, large gatherings with friends and family).

For “individual” contacts (contact type 1), participants were asked about where this contact was made, whether this contact was indoors, outdoors, or both, the duration of this contact, whether this contact involved touch, whether this contact studied or worked at the university (and if so which faculty and school they were associated with), their age, whether they were part of their household, and how often they would usually have contact with this person.

For “other” contacts (contact type 2), no additional questions were asked, as it was expected that there often would be a large number of “other” contacts and participants would not be motivated to answer additional questions about them.

For “group” contacts (contact type 3), participants were asked how many individuals this involved, their ages, whether the majority were from UoB (and if so the main faculty and school this group was associated with), where the group met, whether this was indoors, outdoors or both, whether the members of the group talked to each other and how long the contact with this group was for.

Additionally, participants were asked about symptoms in the last 7 days (listed in Table 5), whether they had sought medical attention for these symptoms, whether they had been self-isolating in the last 7 days, and their COVID-19 status. For some analyses, the variable on whether people have had COVID-19 (no, yes confirmed by a test, yes a doctor suspected so, yes my own suspicions) was combined with the date that they had been tested or were suspected to have COVID-19. This was to create new variables on whether they had COVID-19 in the 2 weeks prior to survey completion, or before this.

Participants who had signed up to repeat questionnaires were sent an email every 8 days with a unique link that allowed their responses to be anonymously connected to those from previous CONQUEST questionnaires that they had responded to. The reminder emails with the survey links were sent every 8 days regardless of whether participants had filled in surveys from previous reminder emails or when they responded to them.

### Analyses

Anonymised data^21^ were downloaded from the REDCap tool and analysed using STATA version 16^22^.

We include records from the 14th September 2020 to 1st November 2020 in order to capture student behaviours at the beginning of term. For some analyses, a comparator population of staff (not including those listed as staff/students) was created taking the same survey dates. We calculated the mean prevalence of behaviours, symptoms, or contacts, stratified by population subgroups.

To investigate the associations between the overall number of contacts on the previous day and demographics and behaviours, univariable and multivariable negative binomial regression modelling was used. These models included variables on: age group (17–24, 25–44, 45–64, 65–79, 80+ years of age), gender (male, female/other—the “other” category had too few individuals and so were grouped with the largest category), under/postgraduate status, current study year (1, 2, 3, 4+), symptoms during the previous week, cardinal symptoms (loss of taste or smell, fever, persistent cough^23^) during the previous week, self-isolating in the prior week, self-reporting being in a high-risk group, household size (1, 2–3, 4–5, 6–9, 10+, missing), and COVID-19 status (never had, previously thought they had it, previously tested positive for it, thought they had it in the last 2 weeks, tested positive for it in the last 2 weeks). Note that all postgraduates were assigned to the 4+ year group to differentiate them from undergraduates in their first year of study. The multivariable models were mutually adjusted for all variables listed.

### Weighting

Initial analyses suggested males and undergraduates were underrepresented in the survey responses. We therefore weighted analyses, with weights based on publicly available UoB data on student demographics, to make the dataset more representative of the university’s student population—see Supplementary Table 1. All tables specify whether weighting was used.

### Ethical approval

Ethical approval was granted on the 14th May 2020 by the Health Sciences University Research Ethics Committee at the University of Bristol (ID = 104,903), with four amendment requests approved on the 22nd May 2020, 9th June 2020, 27th August 2020 and 7th September 2020. The purpose of the amendments was either to update the relevance of the questions or to make the survey faster and easier to complete. All research was performed in accordance with the University of Bristol Ethics of Research Policy and Procedure (http://www.bristol.ac.uk/media-library/sites/red/documents/research-governance/Ethics_Policy_v8_03-07-19.pdf). Participants were aged 18 or older, voluntarily opted-in to the study and were required to give their informed consent before starting the survey.

## Results

### Demographics

From the 14th September 2020 to the 1st November 2020 there were 740 students that completed the questionnaire 1261 times. For a comparator population there were 1655 records from 433 staff.

Most students were aged 17–24, with a median age of 21 (IQR: 19–24) years and a mean age of 23.3 (standard deviation [SD] = 6.8) years. Approximately one quarter (26.2%, 42.5% before weighting) of our student sample were postgraduates aged 25–64. A small proportion (n = 37, 3%) of the students also listed themselves as staff. Just over half (59.3%) of our sample lived in households of 2–5 people. First years had higher mean and maximum household sizes (8.0—SD: 30.4, max: 400) compared to the other years: 4.3 (SD: 2.4, max: 14), 3.9 (SD: 2.5, max: 20), 3.1 (SD: 4.3, max: 60), for years 2, 3, and 4+, respectively (Table 1).

**Table 1:** Unweighted and weighted demographics of the 740 student participants and 1261 student records.

Characteristic	N (%) participants		N (%) records unweighted		N (%) records weighted	
**Age**						
17–24	557	(75.3%)	857	(68.0%)	994	(78.8%)
25–44	168	(22.7%)	368	(29.2%)	225	(19.4%)
45–64	12	(1.6%)	27	(2.1%)	17	(1.4%)
65–79	3	(0.4%)	9	(0.7%)	5	(0.4%)
**Gender**						
Female	520	(70.3%)	868	(68.8%)	675	(53.6%)
Male	207	(28.0%)	368	(29.2%)	564	(44.8%)
Other/prefer not to say	13	(1.8%)	25	(2.0%)	21	(1.7%)
**Ethnicity**						
White	559	(75.5%)	1003	(79.5%)	1004	(79.7%)
Mixed/multiple ethnic groups	33	(4.5%)	57	(4.5%)	56	(4.5%)
Asian/Asian British	117	(15.8%)	163	(12.9%)	160	(12.7%)
Black/African/Caribbean/Black British	7	(1.0%)	7	(0.6%)	6	(0.5%)
Other/prefer not to say	24	(3.2%)	31	(2.5%)	34	(2.7%)
No/don’t know/other	664	(89.7%)	1113	(88.3%)	1137	(90.2%)
Yes	76	(10.3%)	148	(11.7%)	124	(9.8%)
**Student type**						
Undergraduate	474	(64.1%)	725	(57.5%)	931	(73.9%)
Postgraduate	266	(34.0%)	536	(42.5%)	330	(26.2%)
**Year group**						
1	180	(24.3%)	260	(20.6%)	344	(27.3%)
2	122	(16.5%)	205	(16.3%)	247	(19.6%)
3	95	(12.8%)	156	(12.4%)	199	(15.8%)
4+	343	(46.4%)	640	(50.8%)	470	(37.3%)
**Household size**						
1	117	(15.8%)	227	(18.0%)	170	(13.5%)
2–3	245	(33.1%)	449	(35.6%)	430	(34.1%)
4–5	194	(26.2%)	323	(25.6%)	362	(28.7%)
6–9	107	(14.5%)	153	(12.1%)	192	(15.3%)
10+	26	(3.5%)	36	(2.9%)	45	(3.6%)
Unknown	51	(6.9%)	73	(5.8%)	61	(4.8%)
**Residence**						
Catered halls	24	(3.2%)	34	(2.7%)	44	(3.5%)
Self-catered halls	161	(21.8%)	228	(18.1%)	280	(22.2%)
Shared house/flat	349	(47.2%)	613	(48.7%)	642	(51.0%)
Live with family	105	(14.2%)	196	(15.5%)	156	(12.4%)
Live alone	52	(7.0%)	96	(7.6%)	75	(6.0%)
Other	49	(6.6%)	94	(7.5%)	63	(5.0%)

### Symptoms and behaviours

Just over a third of student participants (n = 437, 35%) had experienced symptoms in the week prior to survey, and 93 (7%) had cardinal symptoms, whilst 179 (14%) had been self-isolating in the week prior to the survey (Table 2). Of those with symptoms, 30 (7%) sought medical attention (this could have included: contacting NHS 111, a pharmacist or GP/Practice nurse; visiting a walk-in centre, Accident and Emergency or other hospital). 152 (12%) students thought that they had had COVID-19 (but did not report having had a positive test) more than 2 weeks prior to filling in the survey, whilst 20 (2%) had tested positive more than 2 weeks prior to the survey. 56 (4%) students thought that they had had (but had not tested positive for) COVID-19 within the 2 weeks before completing the survey. 42 (3%) of respondents had tested positive in the 2 weeks prior to survey completion. Students in their first year of study more commonly reported isolating and having cardinal COVID-19 symptoms in the last 7 days before taking the survey, compared to students not in their first year (24% and 15%, respectively), and having tested positive for COVID-19 in the 2 weeks before the survey (10%), than the overall student sample (14% isolating, 7% with cardinal symptoms, and 3%, testing positive).

**Table 2:** Percentage (95% confidence intervals) of student participants isolating within the prior week, with symptoms within the prior week, or suspected of having/testing positive for COVID-19 (all weighted), overall and stratified by study year.

	Study year				Overall
	1	2	3	4 +	
Isolating in the prior 7 days, N = 179	24% (20–29%)	13% (9–18%)	11% (6–15%)	9% (6–11%)	14% (12–16%)
Symptoms in the prior 7 days, N = 437	44% (38–49%)	36% (30–42%)	30% (24–37%)	29% (25–33%)	35% (32–37%)
Cardinal symptoms in the prior 7 days, N = 93	15% (11–19%)	6% (3–9%)	3% (1–6%)	4% (2–6%)	7% (6–9%)
Seeking medical attention for reported symptoms, N = 30	3% (1–5%)	3% (1–5%)	1% (0–2%)	2% (1–3%)	2% (2–3%)
Suspected of having COVID-19 more than 2 weeks before survey*, N = 152	9% (6–12%)	16% (11–20%)	9% (5–14%)	13% (10–16%)	12% (10–14%)
Suspected of having COVID-19 last 2 weeks before survey*, N = 56	5% (3–7%)	8% (4–11%)	6% (3–10%)	2% (0–3%)	4% (3–6%)
Tested COVID positive more than 2 weeks before survey, N = 20	3% (1–5%)	2% (0–4%)	0% (0–1%)	1% (0–1%)	2% (1–2%)
Tested COVID positive last 2 weeks before survey, N = 42	10% (7–13%)	2% (0–4%)	0% (0–0%)	1% (0–1%)	3% (2–4%)

*Medical professional’s opinion or personal suspicion.

Table 3 presents the most common symptoms in the last week reported by students, stratified by their COVID-19 status. All of those that had tested positive in the 2 weeks prior to the survey reported at least one symptom in the prior week but none of these participants reported chilblains, vomiting, or unusual abdominal pain. The most common symptoms among those that had tested positive in the 2 weeks before the survey were a runny nose/sneezing (73%), loss or altered sense of smell (59%), a headache (53%), unusual fatigue (51%), loss or altered sense of taste (49%), and a sore throat (42%). Meanwhile, 36% reported a fever, and 35% a persistent cough; both considered cardinal symptoms of COVID-19.

**Table 3:** Number and percentage of students with symptom type within the week before survey completion, stratified by COVID-19 status.

Symptom	No COVID-19 (N = 992)	Tested positive more than two weeks before survey (N = 20)	Think they have had COVID-19 more than two weeks before survey* (N = 152)	Think they have had COVID-19 within prior two weeks before survey* (N = 56)	Tested positive within the prior two weeks before survey (N = 42)
None	688 (69%)	11 (56%)	96 (63%)	29 (51%)	0 (0%)
Fever	14 (1%)	2 (11%)	6 (4%)	1 (2%)	15 (35%)
Persistent cough	23 (2%)	0 (0%)	2 (2%)	9 (15%)	14 (34%)
Unusual shortness of breath	13 (1%)	5 (26%)	3 (2%)	5 (9%)	3 (8%)
Unusual chest pain or chest tightness	18 (2%)	2 (10%)	2 (2%)	3 (5%)	7 (16%)
Unusual abdominal pain	16 (2%)	2 (11%)	5 (3%)	3 (5%)	0 (0%)
Confusion, disorientation or drowsiness	13 (1%)	0 (0%)	4 (3%)	3 (6%)	4 (8%)
Headache	106 (11%)	1 (5%)	16 (11%)	16 (28%)	22 (52%)
Runny nose/sneezing	157 (16%)	5 (26%)	25 (17%)	13 (23%)	31 (73%)
Unusual fatigue	53 (5%)	0 (0%)	6 (4%)	12 (21%)	21 (51%)
Sore throat	114 (11%)	1 (5%)	17 (11%)	13 (24%)	18 (42%)
Unusual muscle aches	19 (2%)	0 (0%)	6 (4%)	5 (10%)	9 (20%)
Diarrhoea	27 (3%)	3 (15%)	6 (4%)	3 (5%)	5 (11%)
Vomiting	4 (0%)	0 (0%)	1 (1%)	2 (3%)	0 (0%)
Loss or altered sense of taste	1 (0%)	0 (0%)	6 (4%)	6 (11%)	21 (49%)
Loss or altered sense of smell	2 (0%)	0 (0%)	4 (3%)	6 (11%)	24 (58%)
Chilblains on toes or hands	8 (1%)	0 (0%)	4 (3%)	0 (0%)	0 (0%)
Any unexpected rashes	6 (1%)	2 (11%)	0 (0%)	0 (0%)	3 (7%)

*Medical professional’s opinion or personal suspicion.

Those with cardinal symptoms in the week prior to taking the survey were far more likely to have been isolating in that week (61%) than those without these symptoms (11%). 99% of those that had tested positive for COVID-19 during the 2 weeks before survey completion had been isolating within the last week (Table 4). 81% of those that had tested positive for COVID-19 during the 2 weeks prior to the survey had had the cardinal COVID-19 symptoms within the week prior to the survey and 14% of these had sought medical treatment. Of those that suspected that they had had COVID-19 during the 2 weeks prior to the survey but that had not received a positive test, 52% had been self-isolating and 21% reported having the cardinal COVID-19 symptoms within the week prior to the survey.

**Table 4:** Percentage (and standard deviation) of students reporting behaviours or COVID-19 characteristics (weighted), stratified by other behaviours and characteristics.

Group	Isolating in the prior 7 days	Symptoms in the prior 7 days	Cardinal symptoms in the prior 7 days	Sought medical attention for reported symptoms	Suspected having COVID-19 more than 2 weeks before survey*	Suspected having COVID-19 prior 2 weeks before survey*	Tested positive for COVID-19 more than 2 weeks before survey	Tested COVID-19 positive prior 2 weeks before survey
No symptoms within prior week (N = 824)	9% (29%)	0% (0%)	0% (0%)	0% (0%)	12% (32%)	3% (18%)	1% (12%)	0% (0%)
Symptoms within prior week (N = 437)	24% (43%)	100% (0%)	21% (41%)	7% (25%)	13% (33%)	6% (24%)	2% (14%)	10% (29%)
No cardinal symptoms within prior week (N = 1168)	11% (31%)	29% (46%)	0% (0%)	2% (13%)	12% (32%)	4% (19%)	2% (12%)	1% (8%)
Cardinal symptoms within prior week (N = 93)	61% (49%)	100% (0%)	100% (0%)	12% (33%)	13% (33%)	12% (33%)	2% (15%)	36% (48%)
Not had COVID (N = 992)	9% (28%)	31% (46%)	3% (18%)	2% (13%)	0% (0%)	0% (0%)	0% (0%)	0% (0%)
Suspected having COVID-19 more than two weeks before survey* (N = 152)	13% (34%)	36% (48%)	8% (27%)	2% (14%)	100% (0%)	0% (0%)	0% (0%)	0% (0%)
Suspected having COVID-19 prior 2 weeks before survey* (N = 56)	52% (50%)	48% (50%)	21% (41%)	5% (22%)	0% (0%)	100% (0%)	0% (0%)	0% (0%)
Tested positive for COVID-19 more than two weeks before survey (N = 20)	21% (42%)	45% (51%)	10% (31%)	5% (22%)	0% (0%)	0% (0%)	100% (0%)	0% (0%)
Tested positive for COVID-19 prior 2 weeks before survey (N = 42)	99% (11%)	100% (0%)	81% (40%)	14% (35%)	0% (0%)	0% (0%)	0% (0%)	100% (0%)

*Medical professional’s opinion or personal suspicion.

### Contacts

**Figure 1:**
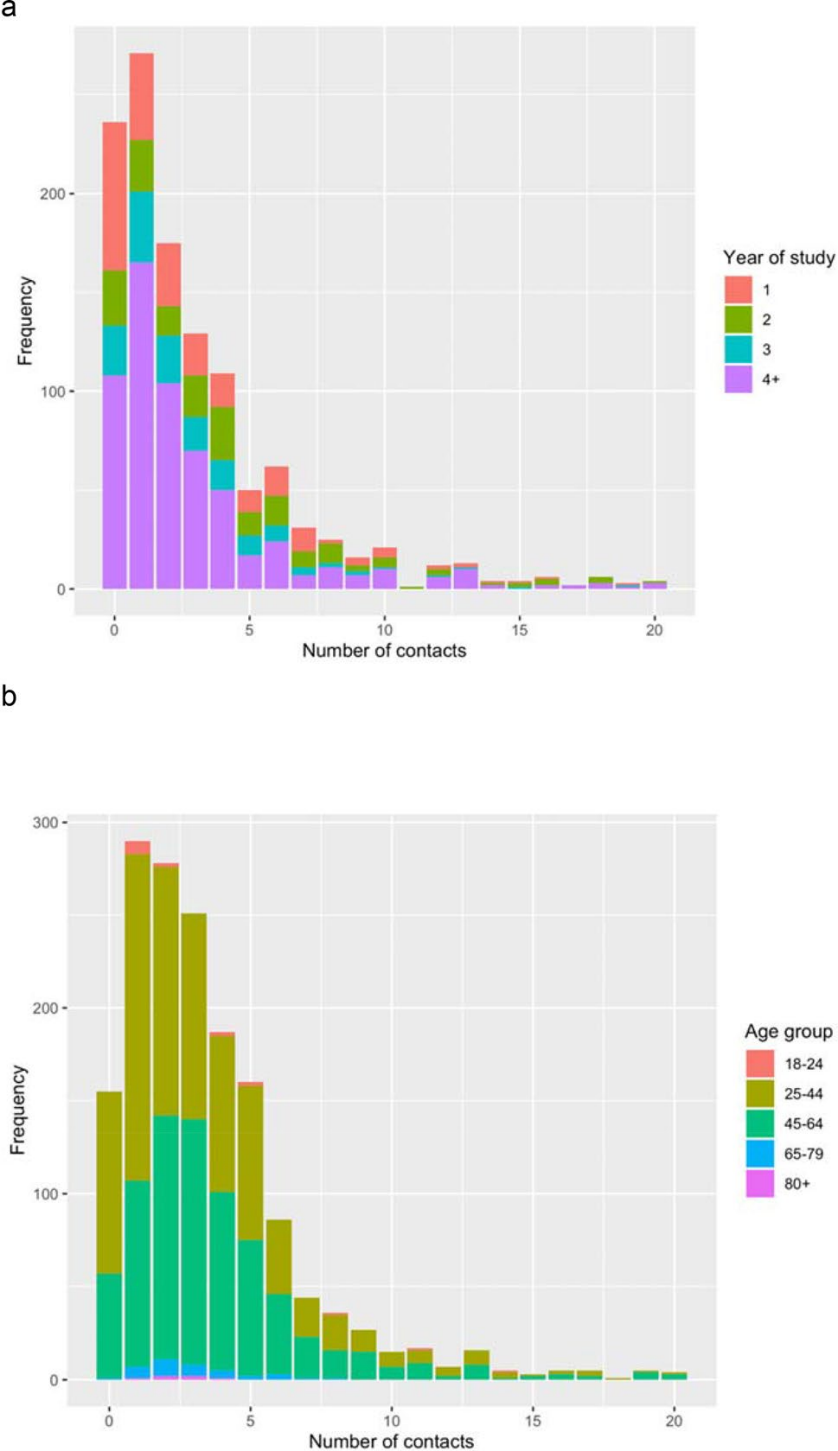
Unweighted histograms of the number of overall contacts* on the previous day among (**a**) students (including staff/students); (**b**) Staff (excluding staff/students). *81 students had more than 20 contacts on the previous day; 58 staff had more than 20 contacts on the previous day—full histograms are shown in Supplementary Figure 1.

The mean number of contacts reported by students for the previous day was 6.1 (SD: 15.0), with a median of 2 (IQR: 1–5). Fewer respondents filled out the survey on Saturdays and Sundays, (10% combined—Supplementary Table 2) compared to weekdays, meaning that data are relatively sparse regarding Fridays and Saturdays. Figure 1 and Supplementary Figures 1–7 show the distribution of the number of contacts on the previous day for students, staff, and various sub-groups of students, as well as different types of contacts. The weighted mean number of responses where participants had 20 or more contacts on the previous day was 8% (SD: 27%). Numbers of contacts reported for the previous day are shown in Supplementary Figure 8, stratified by week. The mean number of contacts appears to be higher from the 5th October onwards; however, there were few survey responses during the first 3 weeks.

Supplementary Table 3 presents a matrix of the mean contacts for the students on the previous day by age-group, with most contacts happening among their own age groups for those aged 18–24 and 25–44. Of the 1261 survey responses, 63 (5%) recorded a contact with someone aged 65 or older, with 27 of these occurring among those aged 17–24, 27 among those aged 25–44, 8 among those aged 45–64, and 1 among those aged 65–79.

**Figure 2:**
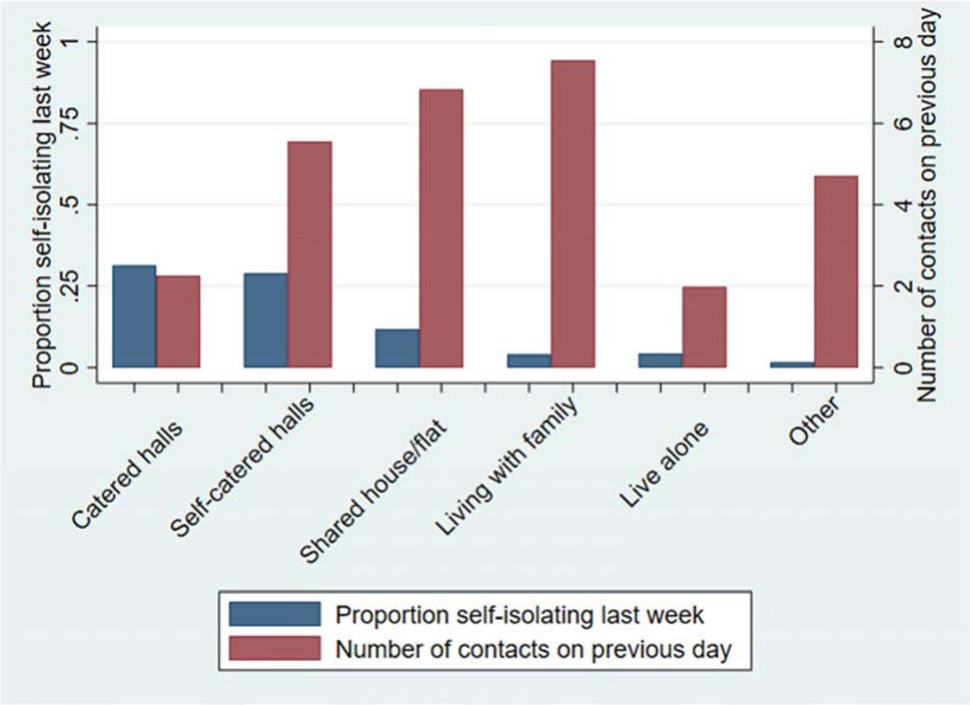
Mean number of contacts on the previous day and the proportion of people self-isolating within the prior week by residence type.

The number of contacts on the previous day and the proportion of participants isolating within the last week by residence type are shown in Fig. 2. Whilst 31% and 29% of those in catered and self-catered halls, respectively, had been isolating within the last 7 days (the majority of which were first years), the mean number of contacts on the previous day appeared higher in the self-catered halls (5.6) than in the catered halls (2.3). Those living in other accommodation types were less likely to have been isolating in the prior week. Participants living with their family appeared to have had the highest mean number of contacts on the previous day (7.5).

Students that reported isolating within the previous week had a lower mean number of contacts on the previous day (4.5) than those not isolating (6.4) (Table 5). The number of "individual" contacts appeared to be similar between those not isolating (2.3) and those isolating (2.1), however the "group" contacts were higher among those not isolating (2.5) than those isolating (1.8), as were "other" contacts (1.6 vs 0.6). Staff had lower mean numbers of overall contacts on the previous day than students (5.2 vs 6.1), which was driven by having lower numbers of "group" (1.8 vs 2.4) and "other" contacts (0.6 vs 1.5).

**Table 5:** Number of contacts types* overall and stratified by isolation status in the last week for students, and overall for staff.

Contact type	Mean (95% confidence interval), median (IQR)			
	Students (weighted)			Staff (unweighted)
	Overall	Not isolating	Isolating	Overall
Overall contacts	6.1 (5.2–6.9), 2 (1–5)	6.4 (5.4–7.3), 2 (1–6)	4.5 (3.0–6.1), 2 (0–5)	5.2 (4.5–5.8), 3 (1.5)
“Individual” contacts	2.2 (2.1–2.4), 2 (1–3)	2.3 (2.2–2.4), 2 (1–3)	2.1 (1.7–2.4), 1 (0.4)	2.8 (2.7–2.9), 3 (1–4)
“Group” contacts	2.4 (2.0–2.8), 0 (0–0)	2.5 (2.0–2.9), 0 (0–0)	1.8 (0.6–3.1), 0 (0–0)	1.8 (1.2–2.3), 0 (0–0)
“Individual and group” contacts	4.6 (4.2–5.1), 2 (1–4)	4.8 (4.3–5.2), 2 (1–5)	3.9 (2.6–5.2), 2 (0–4)	4.6 (4.0–5.2), 3 (1–4)
“Other contacts”	1.5 (0.9–2.1), 0 (0–0)	1.6 (0.9–2.3), 0 (0–0)	0.6 (0.0–1.4), 0 (0–0)	0.6 (0.4–0.8), 0 (0–0)
**Mean (95% confidence interval), median (IQR), % of “individual” contacts (SD)**				
“Individual” contacts	2.2 (2.1–2.4), 2 (1–3)	2.3 (2.2–2.4), 2 (1–3)	2.1 (1.7–2.4), 1 (0–4)	2.8 (2.7–2.9), 3 (1–4)
Contacts with touch	0.8 (0.7–0.8), 0 (0–1), 39% (SD: 41%)	0.8 (0.7–0.8), 0 (0–1), 39% (SD: 41%)	0.8 (0.6–1.0), 0 (0–1), 39% (SD: 44%)	1.4 (1.4–1.5), 1 (1–2), 57% (SD: 36%)
Household member contacts	1.4 (1.3–1.5), 1 (0–2), 64% (SD: 40%)	1.3 (1.3–1.4), 1 (0–2), 61% (SD: 40%)	1.8 (1.5–2.1), 1 (0–3), 84% (SD: 32%)	1.4 (1.3–1.4), 1 (1–2), 57% (SD: 35%)
Frequent contacts (≥ 4 times a week)	1.4 (1.3–1.5) 1 (0–2) 65% (SD: 39%)	1.4 (1.3–1.5), 1 (0–2), 63% (SD: 39%)	1.7 (1.4–2.1), 1 (0–3), 82% (SD: 32%)	1.5 (1.4–1.5), 1 (1–2), 60% (SD: 35%)
**Mean (95% confidence interval), median (IQR), % of “individual and group” contacts (SD)**				
“Individual and group” contacts	4.6 (4.2–5.1), 2 (1–4)	4.8 (4.3–5.2), 2 (1–5)	3.9 (2.6–5.2), 2 (0–4)	4.6 (4.0–5.2), 3 (1–4)
Contacts made at home	1.6 (1.5–1.8), 1 (0–3), 62% (SD: 42%)	1.6 (1.5–1.7), 1 (0–3), 59% (SD: 42%)	2.2 (1.8–2.6), 1 (0–4), 80% (SD: 36%)	1.6 (1.6–1.7), 1 (1–3), 61% (SD: 38%)
Contacts made at university	1.0 (0.8–1.2), 0 (0–0), 10% (SD: 27%)	1.1 (0.8–1.4), 0 (0–0), 10% (SD: 28%)	0.3 (0.1–0.6), 0 (0–0), 7% (SD: 22%)	0.5 (0.3–0.7), 0 (0–0), 7% (SD: 21%)
Contacts made at other location	2.1 (1.7–2.5), 0 (0–1), 33% (SD: 40%)	2.2 (1.8–2.6), 0 (0–1), 35% (SD: 40%)	1.5 (0.4–2.5), 0 (0–0), 18% (SD: 35%)	2.8 (2.2–3.3), 1 (0–2), 38% (SD: 37%)
University of Bristol contactsǂ	3.1 (2.7–3.5), 1 (0–3), 57% (SD: 45%)	3.1 (2.7–3.5), 1 (0–3), 54% (SD: 45%)	3.4 (2.2–4.7), 1 (0–4), 81% (SD: 37%)	0.7 (0.6–0.9), 0 (0–1), 16% (SD: 30%)

ǂThis question asks whether the majority of the group work or study at the University of Bristol. If this was answered “yes”, then we assume here that all members of the group are University of Bristol contacts, if not then we assume that none are.

*“Individual” contacts were the people that the participant spoke to in person one-on-one, including those in the participant’s household and support bubble. “Group” contacts were the contacts that the participant had with large groups of individuals in the same setting (for example, sports teams, tutorials, lectures, religious services, large gatherings with friends and family). “Other” contacts were the many people participants spoke to one-on-one in the same setting where the contacts did not have the opportunity to speak to each other (for example, as part of a customer service role in a shop). Not all of the contact types were asked for each category of contacts, so are only comparable to the associated categories indicated here.

The mean percentage of "individual" contacts on the previous day that involved touch was 39% (SD: 41.0%) overall, 35% (SD: 42%) for males, and 42% (SD: 41%) for females. Overall, the mean percentage of "individual" contacts on the previous day that were with household members was 64% (Table 5). There was a higher percentage of household contacts on the previous day for those who had been isolating within the last 7 days, than for those who had not been isolating within the last 7 days (84% and 61% respectively). Similar results are seen for the percentage of contacts that were frequent (where the person would usually meet that particular contact ≥ 4 times a week) as for those seen for household contacts. 62% of "individual and group" contacts on the previous day were made at the home of the respondent, and this percentage was lower among those not isolating within the last 7 days (59%) than among those that had been isolating (80%). Whilst the percentage of contacts on the previous day made at the university were similar between those that had and had not been isolating within the last 7 days (10% vs 7%), the percentage of contacts at other locations was higher among those that had not been isolating in the prior week (35%) than those that had been isolating (18%). 57% of "individual and group" contacts on the previous day were with other UoB students or staff—this percentage was lower among those not isolating within the past week (54%) than those isolating (81%). In comparison to students, staff had a higher number of contacts on the previous day that involved touch (57% for staff versus 39% for students). Similar numbers of their "individual and group" contacts on the previous day were made at home for staff (61%) and students (62%), whilst far fewer of the contacts of staff on the previous day were either UoB staff or students (16% for staff vs 54% for students). The mean percentage of the student’s “individual” non-UoB contacts that were household members was 50%.

Participants that had not been isolating in the prior week had shorter mean contact durations with their contacts at home (3.3 h) than those that had been isolating (3.9 h), and longer durations of their contacts on the previous day in a location other than home or university (1.1 vs 0.3 h), with both groups have a similar duration of contacts at university (0.2 vs 0.3 h).

In unweighted analyses looking at repeat records from participants, there were 37 records where a participant self-reported not isolating in the 7 days before one survey completion date but then isolating in the 7 days before their next survey completion. For these records, the mean number of contacts was 7.1 (SD: 7.1) for the first survey (when not isolating) and 8.4 (SD: 15.4) at the second (when isolating). There were 20 records where participants went from isolating to not isolating, where the mean number of contacts on the previous day went from 8.7 (SD: 19.6) at the first survey to 9.2 (SD: 13.3) at the second.

There were 17 records where a participant reported a new suspected infection or a positive test within the last 2 weeks, having previously said they had no history of suspected or confirmed infection with COVID-19 (i.e. new cases). For these records, the mean number of contacts on the previous day was 7.8 (SD: 8.2) at the first survey and 6.2 (SD: 6.1) at the second. Only 6 individuals reported current infection, and subsequently reported a previous infection at the next survey. The mean number of contacts reported by these individuals was 3.9 (SD: 4.0) at the first survey and 5.6 (SD: 6.1) at the second.

### Regression analysis

In the multivariable regression analysis of the number of contacts for the previous day (Table 6), older ages were associated with a lower number of contacts when compared with those aged 17–24 years. Students in their 4th (or higher) year of study reported higher numbers of contacts for the previous day than students in their 1st year. Reporting the cardinal COVID-19 symptoms within the last week was associated with a higher number of contacts on the previous day (versus not having the cardinal COVID-19 symptoms), whilst isolating within the week before the survey was associated with having fewer contacts on the previous day.

**Table 6:** Unweighted negative binomial regression analyses (95% confidence intervals [CI]) of number of contacts on the previous day.

Variable	N: mean (SD) contacts	Univariable		Multivariable	
		IRR (95% CI)	*p*-value	IRR (95% CI)	*p*-value
Age 17–24	857: 6.4 (18.3)	Reference	NA	Reference	NA
Age 25–44	368: 4.5 (10.6)	0.71 (0.62, 0.81)	< 0.001	0.54 (0.45, 0.66)	< 0.001
Age 45–64	27: 3.7 (4.4)	0.57 (0.37, 0.88)	0.011	0.29 (0.18, 0.47)	< 0.001
Age 65–79	9: 1.6 (1.0)	0.24 (0.10, 0.56)	0.001	0.34 (0.13, 0.90)	0.029
Female/other	893: 5.5 (15.9)	Reference	NA	Reference	NA
Male	368: 6.5 (16.7)	1.18 (1.03, 1.34)	0.014	1.16 (1.01, 1.35)	0.038
Undergrad	725: 6.0 (11.1)	Reference	NA	Reference	NA
Postgrad	536: 5.5 (21.2)	0.92 (0.81, 1.04)	0.171	0.76 (0.58, 1.00)	0.052
Study year 1	260: 4.4 (8.0)	Reference	NA	Reference	NA
Study year 2	205: 7.5 (10.8)	1.71 (1.40, 2.09)	< 0.001	1.11 (0.84, 1.46)	0.456
Study year 3	156: 4.7 (8.9)	1.08 (0.87, 1.35)	0.480	0.76 (0.56, 1.02)	0.065
Study year 4 +	640: 6.0 (20.7)	1.38 (1.18, 1.62)	< 0.001	1.45 (1.08, 1.95)	0.013
No symptoms	833: 5.6 (17.8)	Reference	NA	Reference	NA
Symptoms	428: 6.2 (12.5)	1.11 (0.98, 1.26)	0.100	1.26 (1.09, 1.45)	0.002
No cardinal symptoms	1186: 5.7 (15.8)	Reference	NA	Reference	NA
Cardinal symptoms	75: 7.3 (21.3)	1.38 (1.00, 1.64)	0.052	1.62 (1.17, 2.24)	0.003
Not isolated last week	1087: 6.0 (16.9)	Reference	NA	Reference	NA
Isolated last week	167: 4.3 (10.6)	0.71 (0.60, 0.85)	< 0.001	0.61 (0.48, 0.76)	< 0.001
Not high risk	1113: (5.8 (16.3)	Reference	NA	Reference	NA
High risk	148: 5.8 (14.9)	1.02 (0.84, 1.22)	0.874	1.00 (0.81, 1.22)	0.984
Household size 1	227: 5.9 (16.9)	1.07 (0.90, 1.28)	0.420	1.24 (1.03, 1.50)	0.026
Household size 2–3	449: 5.5 (11.9)	Reference	NA	Reference	NA
Household size 4–5	323: 7.2 (23.8)	1.31 (1.12, 1.53)	0.001	1.36 (1.15, 1.61)	< 0.001
Household size 6–9	153: 5.7 (9.6)	1.04 (0.85, 1.26)	0.733	1.27 (1.01, 1.59)	0.041
Household size 10 +	36: 3.3 (5.4)	0.60 (0.41, 0.88)	0.010	1.23 (0.78, 1.94)	0.381
Household size missing	73: 1.8 (4.0)	0.33 (0.24, 0.44)	< 0.001	0.49 (0.34, 0.69)	< 0.001
No covid-19	1009: 5.3 (16.3)	Reference	NA	Reference	NA
Previously tested positive more than 2 weeks before survey	14: 8.2 (10.5)	1.54 (0.88, 2.69)	0.130	1.30 (0.73, 2.31)	0.366
Previously suspected to be positive more than 2 weeks before survey	150: 9.2 (19.0)	1.72 (1.43, 2.06)	< 0.001	1.53 (1.26, 1.85)	< 0.001
Suspected to be positive in last 2 weeks	55: 5.4 (8.4)	1.01 (0.75, 1.36)	0.956	1.28 (0.93, 1.76)	0.129
Tested positive in last 2 weeks	33: 2.9 (3.3)	0.55 (0.36, 0.81)	0.003	0.55 (0.33, 0.91)	0.020
Catered halls	34: 2.0 (3.3)	0.32 (0.21, 0.48)	< 0.001	0.34 (0.20, 0.56)	< 0.001
Self-catered halls	228: 4.6 (12.6)	0.73 (0.62, 0.87)	< 0.001	0.68 (0.52, 0.88)	0.003
Shared house/flat	613: 6.2 (11.5)	Reference	NA	Reference	NA
Live with family	196: 8.7 (32.1)	1.41 (1.19, 1.67)	< 0.001	1.84 (1.50, 2.25)	< 0.001
Live alone	96: 2.4 (4.9)	0.38 (0.30, 0.49)	< 0.001	0.74 (0.54, 1.00)	0.051
Other	94: 4.4 (7.5)	0.70 (0.55, 0.89)	0.004	1.06 (0.80, 1.39)	0.698

In the multivariable regression analysis, participants having a household size of 1 was associated with higher numbers of contacts than participants having a household size of 2–3. Similarly, in comparison to having a household size of 2–3, a household size of 4–5 was associated with more contacts, whilst not reporting household size was associated with reporting fewer contacts. COVID-19 status was associated with number of contacts. Those that had not tested positive for or did not suspect themselves to have had COVID-19 had lower numbers of contacts on the previous day than those that suspected themselves to have had COVID-19 more than 2 weeks prior to the survey. Those testing positive within the last 2 weeks before survey completion had fewer contacts. Students in catered and self-catered halls had fewer contacts on the previous day then those living in a shared house/flat but students living in a shared house/flat had fewer contacts than those living with their family. Supplementary Table 4 shows contact numbers stratified by isolation status and under/postgraduate status, with both undergraduates and postgraduates that had been isolating in the previous week having lower numbers of contacts than that had not been isolating.

## Discussion

There has previously been limited quantitative data available on the contacts of university students to inform public health action and mathematical models. Our survey results from the start of the 2020/2021 academic year give insight into the behaviour of university students in this unique and important period in the COVID-19 pandemic, where outbreaks were seen at universities, despite measures being put in place to minimise this risk.

### Contacts

We found a lower mean number of daily contacts among our student population (6.1) than found in surveys carried out before the pandemic (11.7 for adults in Great Britain in the 2004–2008 POLYMOD survey^17^ and 29.9 for students in the 2009 Warwick social contacts survey^14,15^). This result is unsurprising, given the COVID-19 restrictions in place at the time of our survey. Our results on mean number of daily contacts correspond more closely to the CoMix social contacts survey, which has been collecting regular data on contacts from UK adults since early in the pandemic (24th March 2020)^18^. CoMix respondents aged 18–29 had a mean number of daily contacts ranging from 3 to 4.5 from 10th September 2020 to 13th October 2020^24^, while in CONQUEST the mean number of daily contacts ranged from 3 to 6 (Supplementary Figure 8) in the most similar period (14th September 2020 to 26th October 2020).

Despite low numbers of daily contacts being reported by the majority of students (mode = 1, median = 2), there was some heterogeneity in the daily number of contacts, with 8% of students reporting over 20. These individuals may have an increased likelihood of catching COVID-19 and infecting others (so-called “super spreaders”^25^). The Warwick social contacts survey also found a large amount of heterogeneity in number of contacts^14,15^. Theoretical network modelling has shown that disease dynamics can be sensitive towards heterogeneity in contact numbers^26^ and therefore this result could partly explain the outbreak patterns seen at the university during the period studied, although this would need to be confirmed with mathematical modelling.

There were several demographic groups associated with higher numbers of contacts. Students in larger households tended to have more contacts than those in households of sizes 2–3, possibly due to an increased pool of readily available contacts, whilst those in one person households also had higher numbers of contacts than those in 2–3 person households, perhaps because they were required to go out to seek social activities. Students living with their family appeared to report the highest number of contacts, with those living in catered and self-catered halls reporting lower numbers of contacts. Our regression analysis results showed that students in their 4th year of study had higher numbers of contacts than those in year 1, despite living in households with fewer members and adjusting for isolation status. This may be due to students in later years already having established social networks that are less disturbed by the COVID-19 guidelines than the nascent social networks being formed by the first years. It could also be because so many first year students were isolating that this reduced the number of contacts reported by first year students that were not isolating.

When comparing the contacts of students with those of staff, we found that students had slightly higher mean numbers of contacts overall, with the difference driven by having higher numbers of group contacts, possibly due to involvement with university societies, face-to-face teaching (as not all staff are delivering this) and socialising. This corresponds with the POLYMOD survey which found that individuals aged 18–24 (the main age group of students) had more contacts than older adults^17^ However, staff had a higher proportion of contacts involving touch (57%) than students (39%). This could be because students are less likely to live with family members than staff.

Students had most of their contacts at home or university (72%), which was also seen in the Warwick social contacts survey data (82%, 95% confidence interval: 79%-86%). This could suggest that transmission from students to the community is most likely to take place at home and university locations. Students appear to mostly mix with other students, while staff were far less likely to mix with other university staff and students. The POLYMOD survey also found that people of the same age tended to mix with each other^17^. However, around 40% of student contacts in our survey were with people not affiliated to the university, indicating the potential for transmission to groups other than students.

### Isolation behaviour

First year undergraduates were more likely to be isolating within the prior 7 days and to have tested positive for COVID-19 in the prior 2 weeks than other year groups, with higher percentages of respondents isolating that lived in catered and self-catered halls than other accommodation types. This observation confirms that the COVID-19 epidemic among UoB students has been concentrated among first years living in large, shared living residences (as predicted by Brooks-Pollock et al.^13^).

There was high compliance (99%) to isolation guidelines among students who had a positive test for COVID-19 in the previous 2 weeks before survey completion, while half of the students who only suspected they had COVID-19 (but did not have this confirmed by a test) isolated. Some of these students may have been required to isolate due to a member of their household or living circle having a positive test, rather than isolating voluntarily. Just over half of those who reported cardinal symptoms self-isolated, indicating that some students that should have been isolating had not been doing so. This is in contrast to 85% of students who reported that they would self-isolate if they developed coronavirus symptoms in the Office for National Statistic Student COVID-19 Insights Survey pilot run in three English universities from the 12th to the 18th October 2020^27^. The difference in results highlights the discrepancy between intent and action in self-isolation behaviour in students.

Students that had been isolating in the prior week had fewer contacts than those that had not been isolating, with a higher percentage of contacts among those isolating being contacts within their home than for those not isolating. This suggests that whilst the number of contacts of the isolating students was often not as low as might be expected, most contacts that took place were with people they lived with, who were also likely to be isolating.

### Strengths and limitations

The strengths of this survey include the sample size, longitudinal format, and anonymous nature that enable us to capture self-reported behaviours of many students during a key period in the UK's COVID-19 pandemic. In addition, it provides a unique data source on student behaviour during the pandemic, which will be useful in informing public health action and mathematical models. Our results are likely generalisable to other UK city-based universities, as well as to some city-based universities in other countries which are similar in structure and COVID-19 status to UoB. Many of the questions were designed to be comparable to existing contact surveys^14,15,17^.

However, this study has some limitations. Firstly, the number of contacts was asked for the previous day, whilst the questions on self-isolation and symptoms asked about the previous week, and a window of 14-days was used to define current COVID-19 status. This discrepancy in time-windows used for different questions could lead to difficulties in interpreting results, particularly regarding contact patterns for those that had previously been isolating during the prior week but not on the previous day, possibly leading to higher reported contacts for this group. Secondly, the survey questions were devised early in the pandemic when less was known about the epidemiology and possible interventions. We did not capture whether participants had a negative test for COVID-19, which would have been useful information. Thirdly, to capture sufficient detail on contacts, the questionnaire is fairly long (5–10 min) and complicated, which may deter those with many contacts or with little available time from completing the survey, leading to issues with representativeness. Some participants have not filled in their household sizes, which perhaps shows that some people struggled to answer the questionnaires due to the complexity. We included clear instructions defining “contacts” in the survey; however, some people may not read this text or interpret the instructions differently and so there could be variation in what people considered a contact to be.

Selection bias for those who particularly engaged in health-seeking behaviours may have occurred, as those that are less likely to abide by the guidelines may also be less likely to fill out the survey. However, while we are not able to identify the proportion of the population that are not complying with COVID-19 restrictions, we did capture individuals who did not appear to be compliant that were reporting large numbers of contacts and not isolating when experiencing the cardinal symptoms. Another type of selection bias that may have occurred is for students who have had COVID-19. Almost one-fifth of our surveyed student population had tested positive for COVID-19 or suspected that they had had COVID-19, however, only around 7% students had had a positive test as of the 1st November^10^. Nevertheless, the true prevalence of COVID-19 in the student population may be greater than 7% since students with symptoms may not want to present for a test to avoid the potential of obligatory isolation for them and their household. There will inevitably be issues regarding recall bias, particularly when we are asking respondents to estimate when they first think they had COVID-19 (if this hasn’t been confirmed by a positive test), and there will also likely be issues with response bias, leading to inaccurate or false responses.

### Importance and application

Our study comes at a crucial time in the COVID-19 pandemic, Autumn 2020, when the disease was resurgent with high numbers of daily cases, including among university students^7^. It is important to understand the epidemiology of COVID-19 among students due to high transmission rates and their unique mixing patterns, with thousands of young people moving from all over the country and world to study, forming new social networks in the process. Although the student population is mostly young and therefore unlikely to see the worst effects of COVID-19 infection^4,28^, there is the potential for transmission from students back to their families or to other members of the community. Our study is able to provide novel data on student contacts, symptoms, and behaviours at the beginning of the 2020/21 term when several lockdowns of student residences occurred, enabling us to examine adherence to COVID-19 control measures, as well as the outsized influence on the student COVID-19 pandemic of first year undergraduates that mostly reside in very large accommodation blocks with the potential for large scale indoor transmission^13^. We found that the number of daily contacts for students was much lower than in pre-COVID-19 studies, which is likely to be due to the COVID-19 restrictions in place. We show that whilst most students report low numbers of contacts on the previous day, there are a sizeable minority that report large numbers of contacts, highlighting the heterogeneity of transmission and role that individuals with large numbers of daily contacts (potential “super spreaders”) could be having on the spread of disease. Around 40% of student contacts were with people not affiliated to the university, indicating the potential for transmission to groups other than students. This study provides important information for policy makers and mathematical modellers on a key population during the COVID-19 pandemic, as well any future infectious disease outbreaks.

## Supplementary Information

Supplementary Information.

## Data Availability

Data are available at the University of Bristol data repository, data.bris, at 10.5523/bris.2jxe5mx7gzbku2dekvlmbcwwhk, along with the code that was used for the analyses.
